# Choice of crystalloid fluid in the treatment of hyperglycemic emergencies: a systematic review protocol

**DOI:** 10.1186/s13643-019-1130-5

**Published:** 2019-09-03

**Authors:** Benjamin Gershkovich, Shane W. English, Mary-Anne Doyle, Kusum Menon, Lauralyn McIntyre

**Affiliations:** 1 Department of Medicine, University of Ottawa, 401 Smyth Road, Ottawa, Ontario K1H 5B2 Canada; 2 Clinical Epidemiology Program (CEP), Ottawa Hospital Research Institute, 401 Smyth Road, Ottawa, Ontario K1H 5B2 Canada; 3 School of Epidemiology and Public Health, University of Ottawa, Ottawa, Ontario Canada; 4 Ottawa Hospital Research Institute, Ottawa, Ontario Canada; 5 CHEO Research Institute, Ottawa, Ontario Canada; 6 Department of Pediatrics, University of Ottawa, Ottawa, Ontario Canada

**Keywords:** Systematic review, DKA, HHS, Crystalloid fluid, 0.9% saline, Normal saline, Ringer’s lactate, Plasma-Lyte, Hartmann’s, 0.45% saline

## Abstract

**Background:**

Diabetic ketoacidosis (DKA) and hyperglycemic hyperosmolar state (HHS) are life-threatening complications of diabetes mellitus which require prompt treatment with large volume crystalloid fluid administration. A variety of crystalloid fluids is currently available for use and differs in their composition and ion concentrations. While there are potential pros and cons for different crystalloid fluids, it remains unknown if any particular fluid confers a clinical outcome benefit over others in the treatment of hyperglycemic emergencies.

**Methods:**

A systematic search of MEDLINE, Embase, and the Cochrane Library of Systematic Reviews will be conducted to identify eligible studies, which will include observational and interventional studies involving adult and pediatric patients admitted to the hospital with either DKA or HHS. The interventions will include intravenous treatment with 0.9% saline versus other buffered (Ringer’s lactate, Hartmann’s, etc.), and non-buffered (0.45% saline) crystalloid fluids. The primary outcome is mortality at the latest follow-up time point. Secondary outcomes will include mortality at specific time points, length of hospital stay, development of acute kidney injury, requirement for renal replacement therapy, altered level of consciousness, and the time to normalization of several serum biochemical parameters. Where appropriate, meta-analyses will be performed for the outcomes and conducted separately for adult and pediatric patient populations.

**Discussion:**

DKA and HHS are dangerous complications of diabetes mellitus and account for significant morbidity and mortality. Given the importance of crystalloid fluid administration in the management of these conditions, a systematic synthesis of the existing evidence base will identify potential evidence gaps and may help guide future clinical practice.

## Background

Diabetic ketoacidosis (DKA) is an emergent manifestation of diabetes mellitus and is responsible for approximately 30 admissions per 1000 persons with diabetes per year [1]. DKA tends to develop in younger patients with type I diabetes and is characterized by an absolute insulin deficiency with resultant ketone production and acidosis [2]. Hyperglycemic hyperosmolar state (HHS) is a diabetic emergency that shares several common features with DKA but is differentiated by a lack of ketoacidosis [3–5]. Typically seen in older patients with type II diabetes, HHS is often associated with a more profound degree of hyperglycemia and hypovolemia [6]. The in-hospital mortality rate for DKA has decreased over time and is currently less than 1% [1]. In contrast, mortality associated with HHS is nearly tenfold higher [7], potentially due to the higher average age of presentation, as well as greater prevalence of comorbidities in the HHS population [8].

Significant overlap exists between the treatment of DKA and HHS. The approach to both includes crystalloid fluid administration to treat hypovolemia, insulin administration, and close monitoring of electrolytes with replacement as necessary [3, 9]. The degree of serum hyperosmolality observed in DKA and HHS patients is often quite severe and leads to large volume deficits through osmotic diuresis [10]. As a result, patients require several liters of intravenous fluid to correct fluid deficits, restore organ perfusion, maintain hemodynamic stability, and prevent the development of acute kidney injury. In the adult population, this frequently translates to the administration of more than 5 L of fluid over the course of the treatment [11].

The treatment of hyperglycemic emergencies in the pediatric population differs from that of adult populations in several important ways. One additional consideration when treating DKA and HHS in children is the potential for the development of cerebral edema, a rare (< 1%) but potentially fatal complication of pediatric DKA [12]. Several recognized risk factors for developing cerebral edema in pediatric DKA include higher serum urea nitrogen levels, as well as lower arterial carbon dioxide levels at presentation [13]. It has been argued that certain aspects of the treatment of DKA itself, such as the use of hypotonic fluids for resuscitation, may also play a causal role in the development of cerebral edema. However, there remains no consensus about the validity of such claims [14, 15]. A recent multi-center 2 × 2 factorial randomized controlled trial compared 0.45% saline with 0.9% saline in pediatric DKA and found no differences between the two fluids with respect to both short- and long-term neurologic sequelae, defined by altered level of consciousness and intelligence testing up to 6 months after recovery [16].

The need for prompt volume resuscitation in DKA and HHS is reflected in several guidelines for the management of diabetic emergencies. For adult patients with DKA, the American Diabetes Association recommends initial treatment with 1.0–1.5 L of 0.9% saline over 1 h, followed by continuous infusion with either 0.9% or 0.45% saline depending on serum sodium concentration [17]. Similarly, Diabetes Canada recommends initial volume resuscitation with 0.9% saline and suggests varying rates of infusion tailored to the estimated volume deficit of the patient [4]. The guidelines created by the Joint British Diabetes Societies Inpatient Care Group acknowledge the paucity of evidence for use of one type of fluid over another [18]. Nonetheless, they recommend the use of 0.9% saline in the acute management of DKA, given the degree of historical experience with its administration. There are currently no consensus guidelines for the type of fluid or rate of fluid administration for pediatric DKA.

Much interest has been generated in recent years with respect to the choice of crystalloid fluid in acute resuscitation. 0.9% saline, composed of equal concentrations of sodium and chloride, is an inexpensive and commonly used intravenous fluid in the acute setting. However, infusion of 0.9% saline can cause a non-anion gap metabolic acidosis due to the high chloride content in saline and subsequent reduction in serum strong ion difference [19]. Additionally, studies performed in human and animal populations have correlated saline use with renal vasoconstriction, which clinically may translate to a higher risk of acute kidney injury [20, 21].

Similar to 0.9% saline, 0.45% saline is composed of equal concentrations of sodium and chloride ions. However, due to the relative hypotonicity of 0.45% saline, the vast majority of a given volume of infused 0.45% is lost to the extravascular space [22]. While 0.45% saline is rarely used as a resuscitation fluid in adults, several studies have examined its use as both a resuscitative and maintenance fluid in pediatric populations with DKA [16, 23].

Buffered crystalloids, characterized by a lower chloride concentration and the presence of an anion buffer, are on average more expensive than 0.9% saline. However, the more physiologic chloride levels in these fluids generally allows clinicians to avoid the development of hyperchloremic metabolic acidosis. Nonetheless, several potential adverse effects may result from the administration of these fluids. For example, the buffers present in these solutions are converted to bicarbonate upon infusion, which can lead to metabolic alkalosis [24]. The infusion of Ringer’s lactate may also cause elevations in serum lactate levels [25, 26], which may be exaggerated in liver failure and could in turn affect clinical decision-making. The lactate in Ringer’s may be converted to glucose and could exacerbate hyperglycemia in the DKA and HHS setting [27]. Finally, the acetate that is present in Plasma-Lyte and Ringer’s acetate has been associated with altered myocardial activity and hemodynamics in adult patients [28].

Recently, two single institution multiple monthly cross over studies comparing 0.9% saline to balanced crystalloids (Ringer’s Lactate and Plasma-Lyte) conducted in the emergency department (SALT-ED) and intensive care unit (SMART) found small differences in the “Major Adverse Kidney Events within 30 days” composite outcome which included death, requirement for dialysis, or persistent renal dysfunction in favor of balanced crystalloids [29, 30]. These studies did not focus on or specifically describe clinical or biochemical outcomes for the DKA/HHS population.

We argue that the DKA/HHS populations are unique in several ways that make the question of crystalloid fluid choice particularly relevant. Given the association between saline infusion and the development of metabolic acidosis, it would be important to know if 0.9% saline administration has the potential to worsen the already-present acidosis in DKA patients or cause or exacerbate renal injury in the DKA/HHS populations. Alternatively, a large volume administration of buffered and non-buffered crystalloid fluids that are hypotonic in comparison to 0.9% saline (ex: Ringer’s lactate or 0.45% saline) may increase the risk of cerebral edema [31] and confusion, which may be especially relevant in pediatric populations. Furthermore, as highlighted earlier, Ringer’s lactate may also potentiate hyperglycemia in this setting. To address these crystalloid fluid questions, we will perform a systematic review of the literature to determine whether there are differences in clinical outcomes, biochemistries, and endocrine-specific outcomes in patients who are administered 0.9% saline as compared to other buffered and non-buffered crystalloid fluids for the treatment of hyperglycemic emergencies.

## Objectives

The primary objective of this systematic review is to compare 0.9% saline to both buffered crystalloid fluids and other non-buffered crystalloid fluids to determine whether there are differences in overall mortality for patients with DKA or HHS. Secondary objectives will examine differences in in-hospital, 28-day, and 90-day mortality, acute kidney injury and requirement for renal replacement therapy, change in level of consciousness, biochemical disturbances (serum bicarbonate, pH, chloride, anion gap, sodium, glucose), and time to transition from intravenous insulin infusion to subcutaneous insulin.

## Methods/design

This systematic review will be conducted using the principles of the Cochrane Collaboration guide for Systematic Reviews [32] and reported as per PRISMA guidelines [33]. This protocol has been registered with the PROSPERO database for systematic review protocols (Registration ID: CRD42019117866) [34].

### Eligibility criteria

All studies included in this review will be selected in accordance with the PICOS (Population, Intervention, Comparison, Outcomes, Study Design) framework:

#### Population

We will include all patients (pediatric—greater than 1 year of age and less than or equal to 18 years of age—and adult—greater than 18 years of age) with any form of diabetes mellitus, who are admitted to the hospital with a diagnosis of either DKA or HHS as defined in the studies.

#### Intervention

The intervention of interest will be fluid administration with 0.9% saline at any rate.

#### Comparators

0.9% saline will be compared to buffered and other non-buffered crystalloid fluids infused at any rate. Buffered crystalloid fluids will include solutions containing an anion buffer (Ringer’s lactate, Ringer’s acetate, Plasma-Lyte, Hartmann’s solution, etc.). Other non-buffered crystalloid fluids will include 0.45% saline. Dextrose-containing solutions will be excluded as they are only used in the late stages of DKA and HHS management, after normalization of serum glucose [35]. Three percent saline will be excluded as its use in hyperglycemic emergencies is restricted to the treatment of cerebral edema, rather than as a resuscitative or maintenance fluid [36].

#### Outcome measures

We will examine several clinical, biochemical, and endocrine outcomes in the included studies. These specific outcomes were chosen for their overall clinical significance, as well as their particular relevance in the context of DKA/HHS management. For example, treatment duration and length of stay in the hospital for hyperglycemic emergencies may be driven by the time to normalization of several biochemical parameters, including serum bicarbonate and anion gap.

Our primary outcome is the overall mortality measured at the latest follow-up time. Secondary outcomes will include mortality—as measured in-hospital, at 28 days and at 90 days—length of hospital stay, development of acute kidney injury or new requirement for renal replacement therapy, development of altered level of consciousness, time to transition to subcutaneous insulin, and time to normalization of serum bicarbonate, glucose, pH, chloride, sodium, and anion gap (arterial or venous for all serum measurements).

Since DKA and HHS are associated with severe biochemical disturbances which may be exacerbated by crystalloid fluids and contribute to the development of altered level of consciousness (e.g., due to changes in serum osmolality) [35], we will include the Glasgow Coma Scale score as the operational definition for this outcome measure.

#### Study design

Interventional and observational studies will be included. Specifically, we will include randomized and non-randomized controlled trials as well as retrospective and prospective observational studies that compare 0.9% saline to a buffered or other non-buffered crystalloid fluid. Case series and case reports will be excluded, as well as any studies involving non-human subjects.

### Data sources

A literature search will be performed using the MEDLINE and Embase databases, as well as the Cochrane Library of Systematic Reviews. No language or time period limits will be applied in our search. We will hand search references of the primary studies and published narrative and systematic reviews for relevant studies. We will also search ClinicalTrials.gov for unpublished or ongoing clinical trials. Data from conference proceedings will not be searched for this review.

### Search strategy

A search strategy has been designed with the help of a health information specialist with experience in systematic reviews (Appendix). MeSH headings were used in the search strategy to address the relevant aspects of the research question. HHS is often also referred to as hyperglycemic hyperosmolar non-ketotic coma (HONK), and as such, these keywords were also included in the search strategy. Additionally, several different fluids qualify as buffered crystalloid. These include Ringer’s lactate, Ringer’s acetate, Plasma-Lyte, and Hartmann’s solution. All of these keywords were also included in the strategy. No language limits were applied.

### Study selection process

Duplicate citations will be removed from the list of studies generated by the search strategy. After de-duplication, all titles and abstracts will be screened independently by two separate reviewers (BG, BH). Any disagreements will be resolved by a third reviewer (LM). All potentially relevant abstracts will be included for review of the full-text reports with eligibility criteria applied to determine the final number of studies to include in the review.

### Data extraction and management

Data extraction will be performed independently and in duplicate (BG and BH) using a pre-tested electronic data collection form. Data extraction will include information related to study characteristics (i.e., title, authors, year of publication, language, country, journal, study design, sample size, and inclusion/exclusion criteria); population characteristics (i.e., DKA and HHS definitions, pediatric versus adult, age, sex, admission diagnosis, comorbidities, biochemical and physiological parameters measured at baseline including serum bicarbonate, serum glucose, serum pH, serum anion gap, serum creatinine, and Glasgow Coma Scale); interventions and comparators (types of crystalloid resuscitation fluid, fluid protocol details including bolused and infused volumes, length of study period, co-interventions (insulin infusion and subcutaneous insulin protocols); and outcomes (study-specific outcomes as well as our pre-determined primary and secondary outcomes). Different studies may report varying thresholds of “normal” for variables such as serum glucose and bicarbonate; these definitions will be documented in our data abstraction form.

### Data synthesis and analysis

Using tables and text, we will include a summary description of all studies included in the review. 0.9% saline is the control fluid that will be compared against buffered and non-buffered crystalloids for all study outcomes. Primary and secondary outcomes for pediatric and adult studies and for randomized controlled trials and observational studies will be pooled and analyzed separately. We will perform meta-analyses using a random effects model, given the degree of anticipated heterogeneity among included studies [37]. We will calculate statistical heterogeneity among the included studies and use this measure in addition to clinical heterogeneity to inform the appropriateness of data pooling and the conduct of meta-analyses. Statistically, heterogeneity will be expressed using the *I*^2^ statistic and through visual inspection of a funnel plot if there are a sufficient number of trials included [38].

Where appropriate, dichotomous variables will be pooled according to odds ratios (OR) and relative risks (RR) with 95% confidence intervals for observational studies and randomized controlled trials, respectively. With respect to our secondary outcomes, we expect that they will mostly be reported as continuous measures. Continuous variables will be pooled according to mean differences and 95% confidence intervals.

### Subgroup analyses

Given the anticipated clinical heterogeneity among the included studies, we will perform subgroup analyses to examine differences in the primary and secondary outcomes for DKA and HHS patients, for patients with pre-existing chronic kidney disease according to definitions used in the included studies, for patients who received an initial bolus of crystalloid for resuscitation, and for patients with severe DKA. Severe DKA will be defined using pre-specified criteria [39]. We will also examine differences according to specific buffered and other non-buffered crystalloid fluids (ex 0.9% saline versus Ringer’s lactate, 0.9% saline versus Ringer’s acetate, 0.9% saline versus Hartmann’s, 0.9% saline versus 0.45% saline).

### Sensitivity analysis

Sensitivity analyses according to randomized controlled trials and observational studies that are considered low risk of bias across all domains will examine the robustness of the treatment effect on the primary outcome.

### Assessment of methodological quality

Risk of bias for each of the included studies will be assessed using the Cochrane Collaboration tool for assessing the risk of bias [40] for RCTs and the Newcastle-Ottawa scale for non-randomized trials [41]. Where appropriate, the GRADE system [42] will be used to rate the quality of evidence for each of the reported outcomes.

## Discussion

DKA and HHS are life-threatening complications of diabetes mellitus and account for significant morbidity and mortality. Although it has been known for decades that crystalloid fluid administration is a cornerstone of the treatment for both conditions, it remains unclear if any particular type of crystalloid resuscitation fluid is superior to another in hyperglycemic emergencies. This systematic review will provide a comprehensive summary of the clinical, biochemical, and endocrinologic evidence to inform clinical practice, identify research gaps, and guide future crystalloid resuscitation fluid research questions for the treatment of adults and children with DKA and HHS.

## Data Availability

Not applicable.

## Appendix

### Search strategy

Database: Embase Classic+Embase <1947 to 2018 September 27>, Ovid MEDLINE(R) ALL <1946 to September 27, 2018>, EBM Reviews - Cochrane Central Register of Controlled Trials <August 2018>.

Search Strategy:

1. ringer*.tw,kw. (33841)
2. (hartmann* adj5 solution).tw. or hartmann*.kf. (726)
3. ((buffer* or balance*) adj5 (crystalloid* or solution)).tw. (39601)
4. Plasma-Lyte.tw,kw. (383)
5. PlasmaLyte.tw,kw. (395)
6. (Isotonic Solutions/ or Fluid Therapy/) and (buffer* or balance*).tw,kw. (3677)
7. sodium lactate.tw,kw. (2414)
8. ((buffer* or balance*) and (crystalloid* or solution)).kf. (266)
9. (half adj3 (saline or sodium chloride or nacl)).tw. (1045)
10. (half saline or half sodium chloride or half nacl).kw. (1)
11. Sodium Chloride/ and (half or “0.45”).tw,kf. (6320)
12. (“0.45%” and (saline or nacl or sodium chloride)).tw. (3267)
13. or/1-12 (87112)
14. Diabetic Ketoacidosis/ (17580)
15. Hyperglycemic Hyperosmolar Nonketotic Coma/ (1254)
16. (Hyperosmolar hyperglycemi* or Hyperosmolar hyperglycaemi*).tw,kw. (649)
17. Ketoacidosis*.tw,kw. (17702)
18. (diabetic acidosis or diabetic ketosis or dka).tw,kw. (6604)
19. or/14-18 (26914)
20. 13 and 19 (201)
21. exp. animals/not humans/(16932608)
22. 20 not 21 (147)
23. 22 use medall (60)
24. calcium chloride plus potassium chloride plus sodium chloride/ (78)
25. Ringer lactate solution/ (6850)
26. isotonic solution/ or Ringer solution/ (18262)
27. Hartmann solution/ (565)
28. ((hartmann* adj5 solution) or ringer*).tw. (34349)
29. ((buffer* or balance*) adj5 (crystalloid* or solution)).tw. (39601)
30. acetic acid plus gluconate sodium plus magnesium chloride plus potassium chloride plus sodium chloride/ (280)
31. (plasmalyte or plasma lyte).tw. (757)
32. (fluid therapy/ or fluid resuscitation/) and (buffer* or balance*).tw. (2933)
33. lactate sodium/ (1966)
34. lactate sodium.tw. (214)
35. (half adj3 (saline or sodium chloride or nacl)).tw. (1045)
36. (half saline or half sodium chloride or half nacl).kw. (1)
37. Sodium Chloride/ and (half or “0.45”).tw. (6320)
38. (“0.45%” and (saline or nacl or sodium chloride)).tw. (3267)
39. or/24-38 (100550)
40. diabetic ketoacidosis/ (17580)
41. (ketoacidosis or diabetic acidosis or diabetic ketosis or dka).tw. (19587)
42. nonketotic diabetic coma/ (602)
43. (Hyperosmolar hyperglycemi* or Hyperosmolar hyperglycaemi*).tw. (627)
44. or/40–43 (26316)
45. 39 and 44 (326)
46. (exp animal/ or nonhuman/) not exp. human/ (11182888)
47. 45 not 46 (310)
48. 47 use emczd (217)
49. ringer*.tw,kw. (33841)
50. (hartmann* adj5 solution).tw. or hartmann*.kw. (1107)
51. ((buffer* or balance*) adj5 (crystalloid* or solution)).tw. (39601)
52. Plasma-Lyte.tw,kw. (383)
53. PlasmaLyte.tw,kw. (395)
54. (Isotonic Solutions/or Fluid Therapy/) and (buffer* or balance*).tw,kw. (3677)
55. sodium lactate.tw,kw. (2414)
56. ((buffer* or balance*) and (crystalloid* or solution)).kw. (445)
57. (half adj3 (saline or sodium chloride or nacl)).tw. (1045)
58. (half saline or half sodium chloride or half nacl).kw. (1)
59. Sodium Chloride/and (half or “0.45”).tw,kf. (6320)
60. (“0.45%” and (saline or nacl or sodium chloride)).tw. (3267)
61. or/49-60 (87457)
62. Diabetic Ketoacidosis/ (17580)
63. Hyperglycemic Hyperosmolar Nonketotic Coma/(1254)
64. (Hyperosmolar hyperglycemi* or Hyperosmolar hyperglycaemi*).tw,kw. (649)
65. Ketoacidosis*.tw,kw. (17702)
66. (diabetic acidosis or diabetic ketosis or dka).tw,kw. (6604)
67. or/62-66 (26914)
68. 61 and 67 (202)
69. 68 use cctr (8)
70. 23 or 48 or 69 (285)
71. remove duplicates from 70 (228)
72. **71 use medall (60) Medline**
73. **71 use emczd (166) Embase**
74. **71 use cctr (2) Cochrane**

## Abbreviations

DKA: Diabetic ketoacidosis
HHS: Hyperglycemic hyperosmolar state
HONK: Hyperglycemic hyperosmolar non-ketotic coma
OR: Odds ratio
PICOS: Population, intervention, comparison, outcomes, study design
RR: Relative risk
